# Supplementary material for: Evidence of Bacterial Community Coalescence between Freshwater and Discharged tpm-Harboring Bacterial Taxa from Hospital and Domestic Wastewater Treatment Plants among Epilithic Biofilms
Source: Microorganisms. 2023 Apr 2;11(4):922. doi: 10.3390/microorganisms11040922 (PMC10144666; doi:10.3390/microorganisms11040922)
Supplement: Supplementary file 1 [file microorganisms-11-00922-s001.zip › Bouchali et al Supplementary Figures R2.pdf]

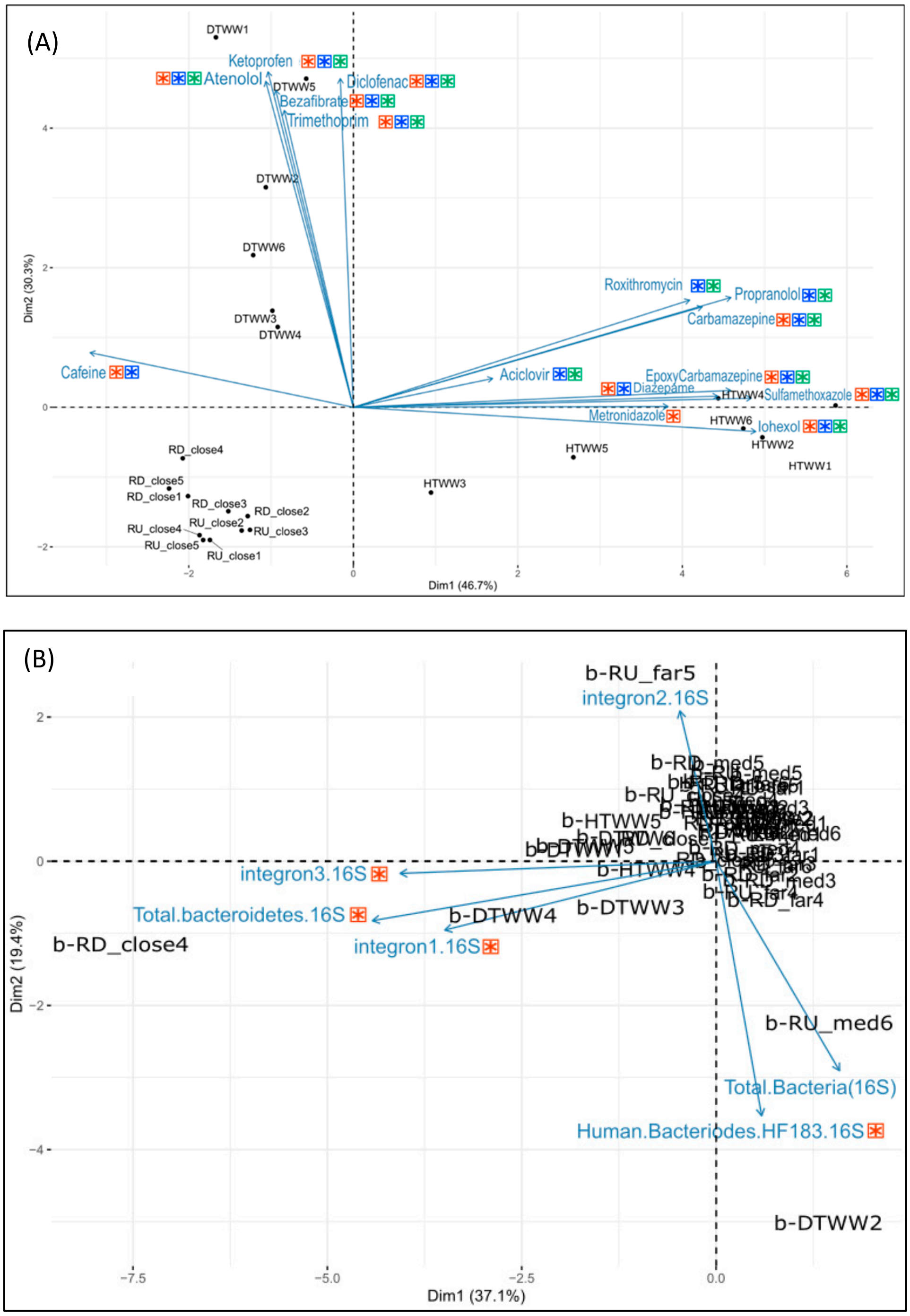

Figure S1. Principal component analyses (PCA) computed on the dataset of (A) 15 pharmaceuticals monitored from the HTWW, DTWW and river water samples and (B) microbiological parameter monitored among all the blueschist rock biofilm (b-) samples (**Table S1**). Ordinations of the HTWW, DTWW, and river compartments according to these monitored parameters are shown. b-: biofilm; HTWW: hospital sewer treated wastewaters; DTWW: domestic sewer treated wastewaters.

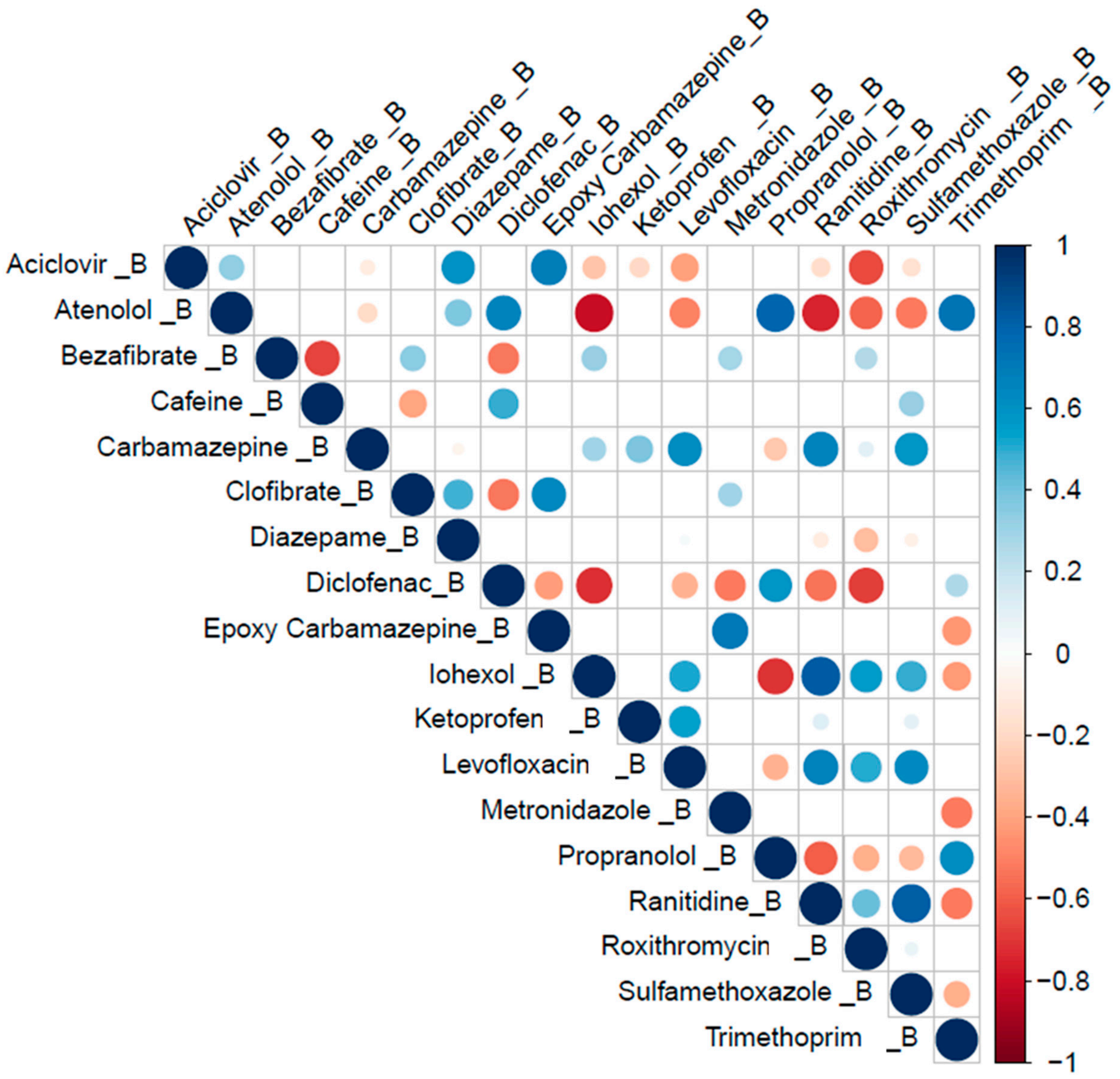

Figure S2a. Correlogram illustrating the significance of the Spearman correlation tests between the pharmaceutical concentrations monitored in b-DTWW and b-HTWW biofilm samples (**Table S1**). All presented correlations had a p-value < 0.05. b-HTWW: rock biofilms generated from hospital sewer treated wastewaters; b-DTWW: rock biofilms generated from domestic sewer treated wastewaters.

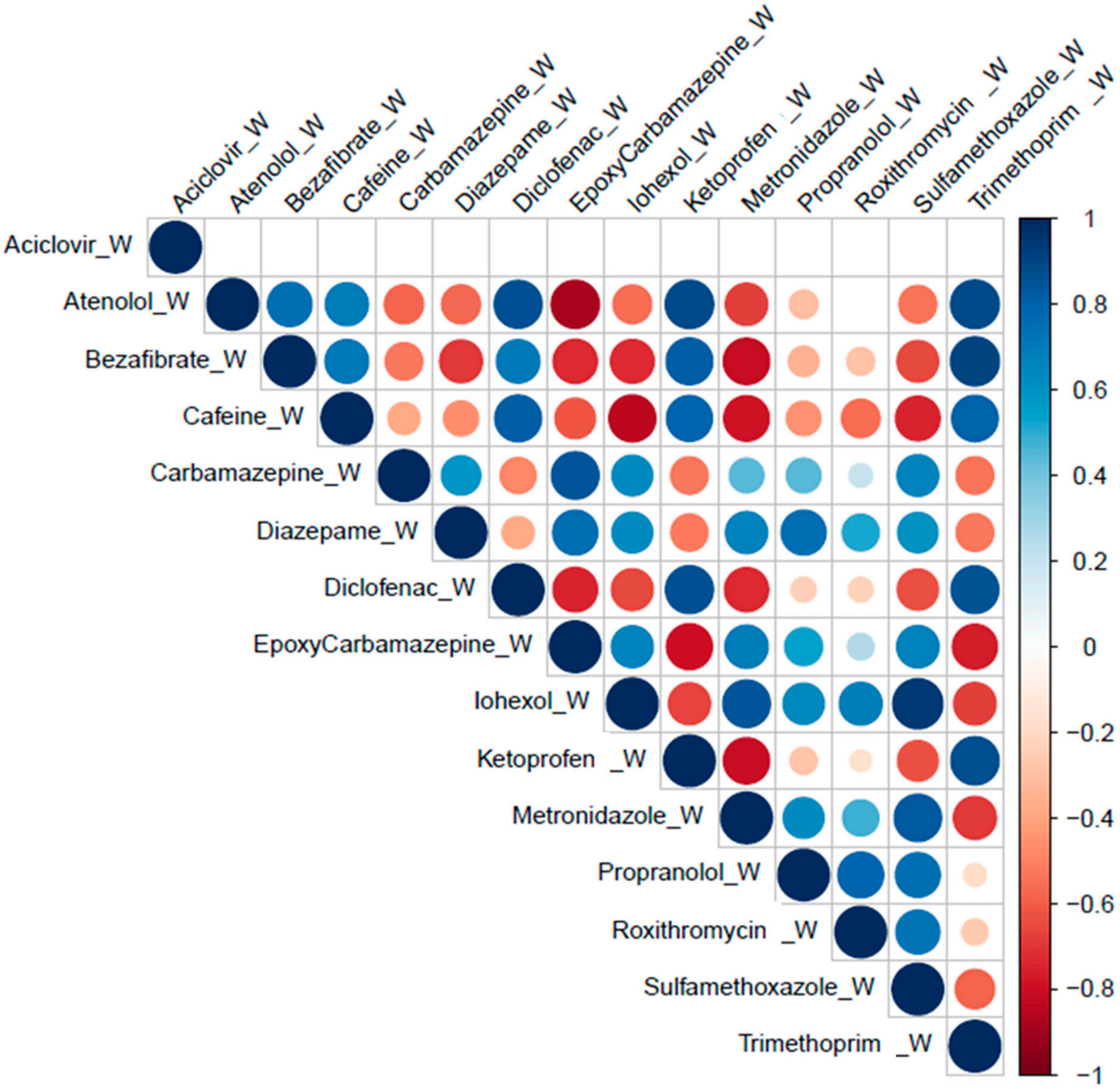

Figure S2b. Correlogram illustrating the significance of the Spearman correlation tests between the pharmaceutical concentrations monitored in DTWW and HTWW water samples (**Table S1**). All presented correlations had a p-value < 0.05. HTWW: hospital sewer treated wastewaters; DTWW: domestic sewer treated wastewaters.

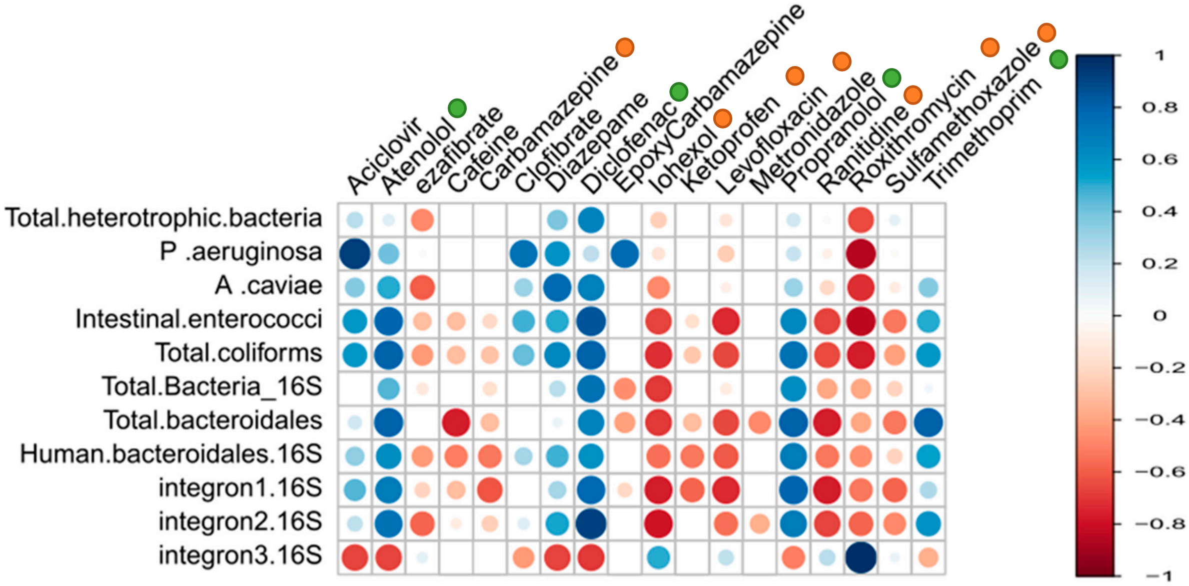

Figure S3. Correlogram illustrating the significance of the Spearman correlation tests performed between the pharmaceutical concentrations and the microbiological parameters recorded among the b-DTWW and b-HTWW biofilm samples (**Table S1**). All presented correlations had a p-value < 0.05. Orange and green circles by the name of the monitored pharmaceuticals indicate substances restricted to respectively HTWW and DTWW biofilm samples according to **Figure 2**. *E. coli* was not considered in these analyses because of missing values. b-HTWW: rock biofilms generated from hospital sewer treated wastewaters; b-DTWW: rock biofilms generated from domestic sewer treated wastewaters.

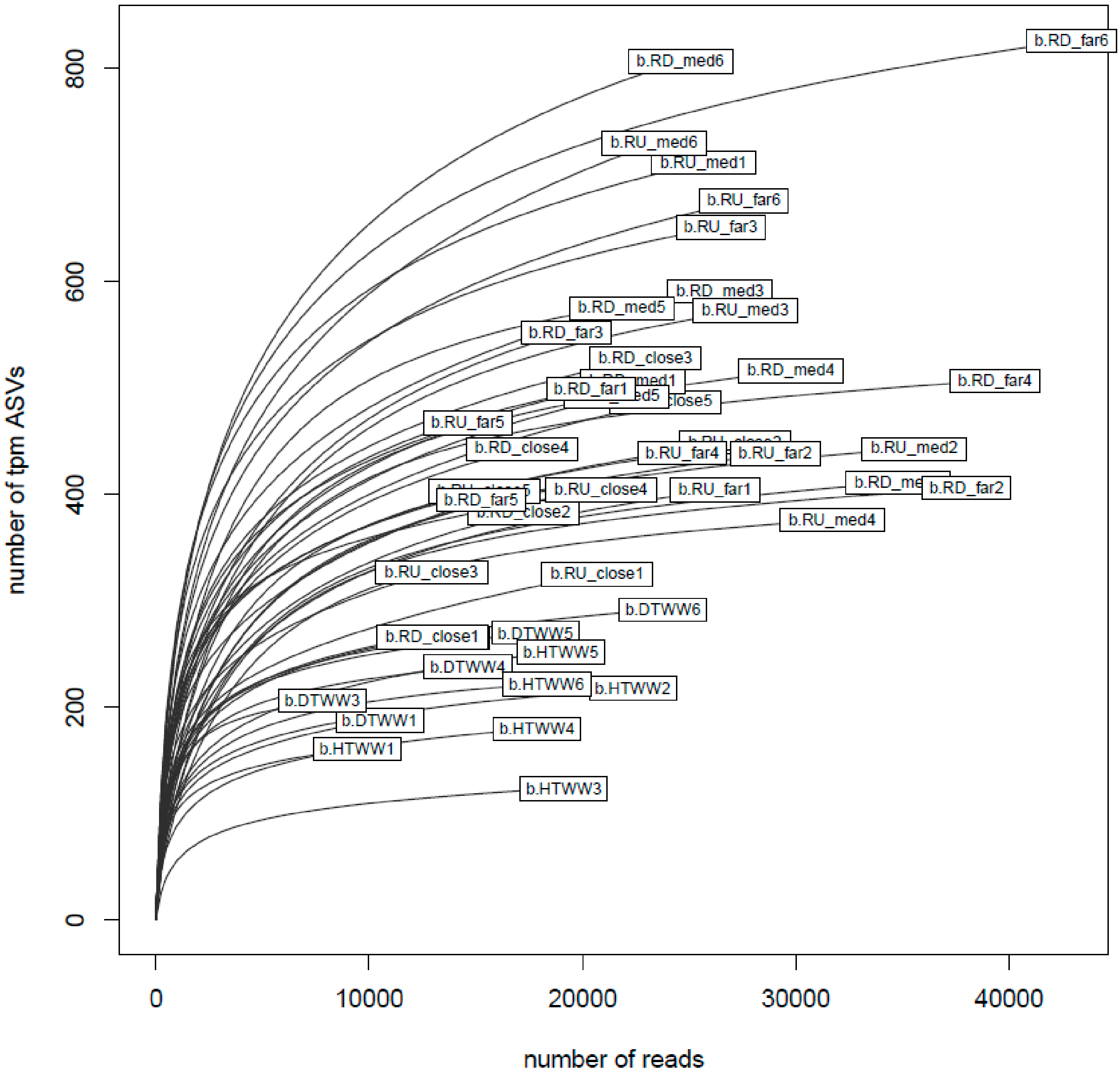

Figure S4. Rarefaction curves showing the numbers of *tpm* gene reads according to the *tpm* ASV numbers per biofilm samples exposed to hospital sewer treated wastewaters (b-HTWW), domestic sewer treated wastewaters (b-DTWW), river surface waters upstream (b-RU) or downstream (b-RD) the outlet of the WWTP at a close, medium (med) or large distance according to Figure 1.
